# Effect of grain size on thermal transport in post-annealed antimony telluride thin films

**DOI:** 10.1186/s11671-015-0733-6

**Published:** 2015-01-28

**Authors:** No-Won Park, Won-Yong Lee, Ji-Eun Hong, Tae-Hyun Park, Soon-Gil Yoon, Hyunsik Im, Hyung Sang Kim, Sang-Kwon Lee

**Affiliations:** Department of Physics, Chung-Ang University, Seoul, 156-756 Republic of Korea; Department of Materials Engineering, Chungnam National University, Daejeon, 305-764 Republic of Korea; Department of Semiconductor Science and Physics, Dongguk University, Seoul, 100-715 Republic of Korea

**Keywords:** Thermal conductivity, Antimony telluride (Sb_2_Te_3_), 3-*ω* technique, Grain size, Thermal transport

## Abstract

The effects of grain size and strain on the temperature-dependent thermal transport of antimony telluride (Sb_2_Te_3_) thin films, controlled using post-annealing temperatures of 200°C to 350°C, were investigated using the 3-omega method. The measured total thermal conductivities of 400-nm-thick thin films annealed at temperatures of 200°C, 250°C, 300°C, 320°C, and 350°C were determined to be 2.0 to 3.7 W/m · K in the 20 to 300 K temperature range. We found that the film grain size, rather than the strain, had the most prominent effect on the reduction of the total thermal conductivity. To confirm the effect of grain size on temperature-dependent thermal transport in the thin films, the experimental results were analyzed using a modified Callaway model approach.

## Background

Thermoelectric (TE) materials and devices have been widely investigated in recent decades [1-3], and TE devices have been notably applied in solid-state refrigeration and power conversion. In general, the performances of these TE devices in terms of energy conversion from heat to electricity can be determined simply from the dimensionless figure-of-merit, ZT [4-6], which is defined as ZT = *S*^2^*σT*/*κ*, where *S*^2^*σ* is the power factor, *S* is the Seebeck coefficient, *σ* is the electrical conductivity, *T* is the absolute temperature, and *κ* is the thermal conductivity. To achieve high ZT values, TE devices require both high power factors and high electric conductivity, along with low thermal conductivity. Based on these properties, the reduction of thermal conductivity with the retention of a high power factor is thought to be the most effective approach toward improving TE performance. In particular, it is notable that two-dimensional (2D) thin-film-based TE devices have led to new opportunities for solid-state microelectronic applications by enabling the integration of TE cooling devices into microelectronic systems [6]. In addition, they can overcome the limitations of 1D materials with regard to dimensionality, morphology, and application to scalable large-area devices, despite the fact that 1D nanomaterials have significantly lower thermal conductivities compared to higher dimensional materials, including bulk materials, at room temperature [7-10]. Recently, Venkatasubramanian et al. reported that Bi_2_Te_3_/Sb_2_Te_3_ superlattice thin films prepared using metal-organic chemical vapor deposition have exhibited a ZT value of approximately 2.4 at room temperature, which clearly indicates a significant improvement in TE device performance compared to that of the state-of-the-art bulk alloy at room temperature [6]. Additionally, nanostructured Bi-, Sb-, Te-, and Se-based thin films, such as Bi_2_Te_3_ [11], Bi_0.5_Sb_1.5_Te_3_ [12], Pb-doped Bi_2_Te_3_ [13], Bi_2_Se_3_ [14], Sb_2_Te_3_, Bi_2_Te_3_/Sb_2_Te_3_ [15-17], Bi_0.5_Sb_1.5_Te_3_, and Bi_2_Te_2.7_Se_0.3_ [18], have been proven to be promising TE materials at room temperature.

Antimony telluride (Sb_2_Te_3_) is a narrow-band-gap (approximately 0.2 eV) semiconductor with a tetradymite structure and has been shown to be one of the most promising TE materials at room temperature [19]. Das et al. have studied the TE and electrical properties of crystalline Sb_2_Te_3_ thin films as a function of temperature and film thickness, including thermal power, the Seebeck coefficient, and electric resistivity [15]. They have reported that both the TE power and electrical resistivity are linear functions of the reciprocal of the film thickness. Furthermore, while many previous works have primarily examined the electrical properties and power factor of Sb_2_Te_3_, including the Seebeck coefficient [15,20-22], only a small number of works on the temperature-dependent thermal conductivity of Sb_2_Te_3_ thin films have been reported, despite the fact that this substance has been proven to be an excellent TE material. Moreover, the effects of the nanostructures and morphologies of Sb_2_Te_3_ thin films, including the strain and grain size, on the temperature-dependent thermal transport are also important with regard to further understanding of the thermal properties of these films. This is especially true since grain size and strain may play a significant role in thermal transport [23]. For example, it has been reported that the effects of strain in TE materials enhance the thermal performance, since the thermal conductivity increases as the compressive strain increases, while it decreases with increasing tensile strain [24,25].

In this study, Sb_2_Te_3_ thin films were deposited at room temperature using RF magnetron sputtering, and post-annealing treatments were then employed at 200°C, 250°C, 300°C, 320°C, and 350°C for 5 min under an Ar atmosphere to enhance the TE properties of the resultant films. We investigated the influence of the post-annealing process on the structure, chemical composition, and thermal transport of the Sb_2_Te_3_ thin films. In particular, the temperature-dependent thermal conductivity was measured in the temperature range of 20 to 300 K using the four-point-probe 3-*ω* method. A theoretical model study using the modified Callaway model is also reported here, which was conducted to further investigate the effects of strain and grain size on the thermal transport of the films.

## Methods

### Sample preparation

Four-hundred-nanometer-thick Sb_2_Te_3_ thin films were prepared on a SiO_2_ (300 nm thick)/Si (001) substrate at room temperature using RF magnetron sputtering with a highly pure Sb_2_Te_3_ as a target (99.99% purity). The detailed deposition process has been described elsewhere [26]. In brief, to tune the film nanostructures, post-annealing processes were performed at temperatures of 200°C, 250°C, 300°C, 320°C, and 350°C for 5 min under an Ar atmosphere of approximately 1.0 × 10^5^ to 1.0 × 10^−2^ Pa. The properties of the film crystal structures, including crystal orientation, average grain size, and lattice parameters, were analyzed using X-ray diffraction (XRD; Rigaku O/MAX-RC, Rigaku, Shibuya-ku, Japan), while the surface morphologies of the films were characterized using a field emission scanning electron microscope (FE-SEM; SIGMA/Carl Zeiss, Seoul, South Korea) equipped with energy dispersive X-ray spectrometry (EDX). The in-plane electrical conductivity of the films was measured at room temperature using a four-point probe method, and the out-of-plane (cross-plane) thermal conductivity (*κ*_f_) of the thin film was measured using a four-point-probe differential 3-*ω* technique with an accuracy of ±5% [27]. A detailed description of the measurement setup can be found in our previous publication [28]. Briefly, a thin SiO_2_ layer (approximately 100 nm) was first deposited onto the Sb_2_Te_3_ thin film through plasma-enhanced chemical vapor deposition to ensure the electrical insulation of the films. Narrow four-point probe metal electrodes composed of Ti/Au (10 nm/300 nm), in which the metal electronics act as both heaters as well as sensors for measuring the temperature gradient, were then patterned onto the films using a photolithography process.

### Thermal conductivity measurement using differential 3-ω method

The thermal transport measurements were performed in the 20 to 300 K temperature ranges in a closed-cycle refrigerator (CCR; Janis, Woburn, USA) system equipped with a turbo pump (Edwards, North Somerset, UK). In the differential 3-*ω* method, the total temperature oscillation, Δ*T* (*ω*), for a multilayer sample can be given by [29]

1 $$ \varDelta T\left(\omega \right)=\frac{P}{\pi {\kappa}_s}\left\{\frac{1}{2} ln\left(\frac{D_s}{b^2}\right)+0.923-\frac{1}{2} ln\left(2\omega \right)-\frac{i\omega }{4}\right\}+\frac{P{d}_f}{2b{\kappa}_f}, $$

where *P* is the supplied power-per-unit-length of the narrow metal line; *D*_s_ is the thermal diffusivity; *d*_f_ is the thin film thickness; *b* is the width; and *κ*_s_ and *κ*_f_ are the thermal conductivities of the SiO_2_(300-nm-thick)/Si substrate and 400-nm-thick Sb_2_Te_3_ thin film, respectively. Δ*T* (*ω*) is obtained from measurements of the third-harmonic root-mean-square voltage drop, *V*_rms-3ω_, across the metal line, using the following equation:

2 $$ \Delta T\left(\omega \right)=\frac{2{V}_{rms-3\omega }}{\alpha {I}_0{R}_0},\kern0.24em \alpha =\frac{1}{R_0}\left(\frac{d{R}_0}{dT}\right) $$

Here, *α* is the temperature coefficient of the resistance, *R*_0_, of the Ti/Au metal strip. Finally, *κ*_f_ is determined using Equation 3, which can be derived from the second term in Equation 1, such that

3 $$ {\kappa}_f=\frac{P{d}_f}{2b\left\{\Delta {T}_{s+f}\left(\omega \right)-\Delta {T}_s\left(\omega \right)\right\}}, $$

where Δ*T*_s+f_ (*ω*) is the temperature oscillation of the in-phase component for the SiO_2_/Si substrate with the thin film and Δ*T*_*s*_ (*ω*) is the temperature oscillation of the in-phase component without the thin film. Thus, the out-of-plane thermal conductivity of the thin films can generally be evaluated using Equation 3, provided Δ*T*_s+f_ (*ω*) and Δ*T*_*s*_ (*ω*) are measured separately using the 3-*ω* method in the 20 to 300 K temperature range.

## Results and discussion

### Material characteristics of Sb_2_Te_3_ thin film

**Figure 1:**
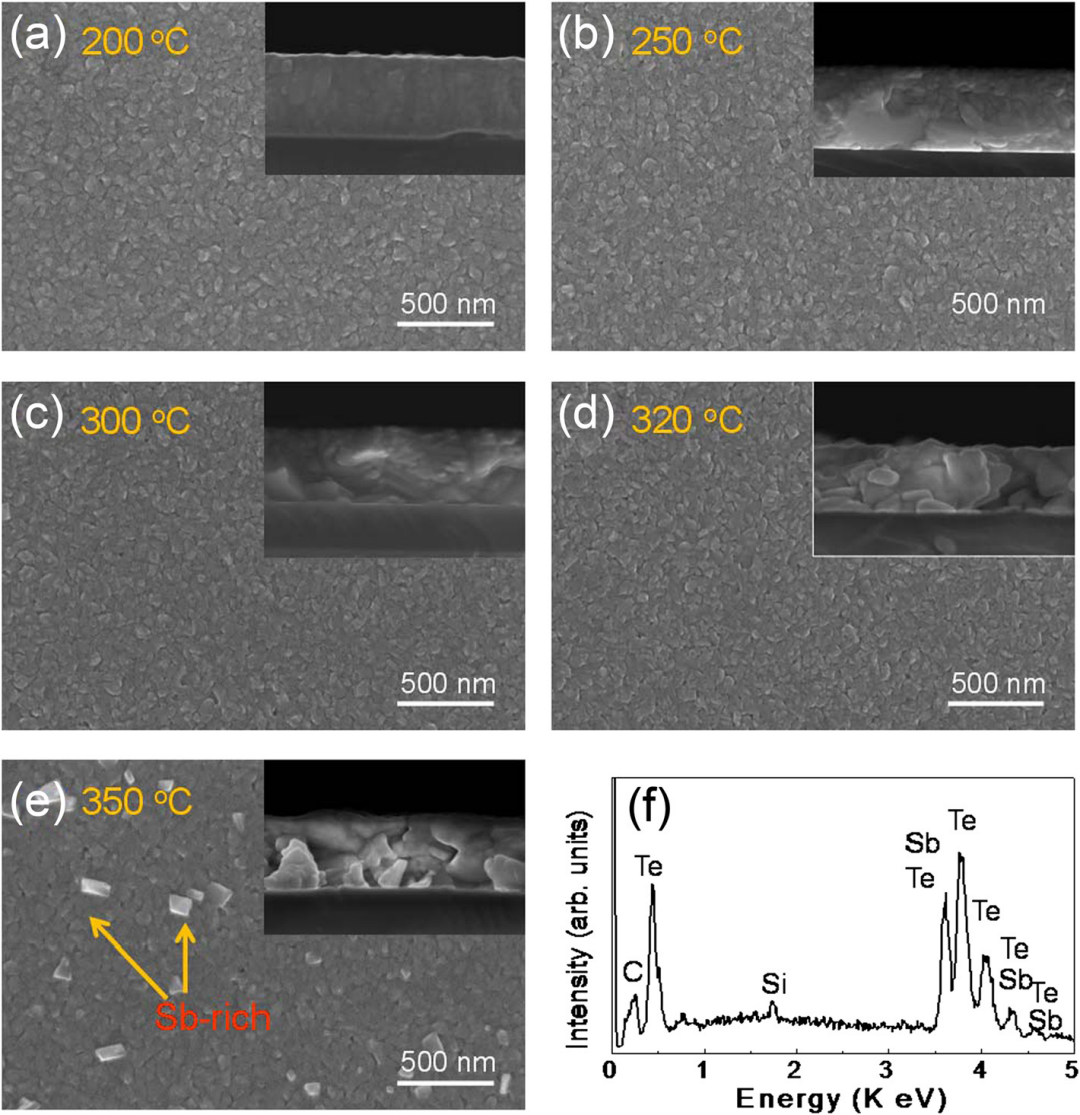
**Surfaces and cross-planes of the films annealed at 200°C, 250°C, 300°C, 320°C, and 350°C. (a-e)** SEM surface images (top-view images) of the Sb2Te3 thin film of 400-nm thickness with post-annealing temperatures of 200°C, 250°C, 300°C, 320°C, and 350°C, respectively. The insets in each figure show cross-sectional images (tilted-view images) of the films annealed at temperatures of 200°C to 350°C. **(f)** EDX spectra of Sb_2_Te_3_ thin film at room temperature.

First, the surface morphologies (including grain size and boundary and atomic compositions) of the Sb_2_Te_3_ thin films were investigated using FE-SEM with EDX. Figure 1a,b,c,d,e shows the surfaces and cross-planes (inset) of the films annealed at 200°C, 250°C, 300°C, 320°C, and 350°C, respectively. These images clearly reveal the effect of the post-annealing temperature on these characteristics (Figure 1). As shown in Figure 1, the grains and grain boundary of all the annealed films are uniform, even though Sb-rich crystalloid precipitates appeared on the surfaces of the films annealed at 350°C. This crystalloid precipitate was confirmed by EDX measurement (Figure 1f). Previous works have also reported these Sb-rich precipitates in annealed Bi-Sb-Te [30,31] and in annealed Sb_2_Te_3_ films [16]. Moreover, we examined the stoichiometry of the annealed Sb_2_Te_3_ thin films using EDX measurements, as shown in Figure 1f. From the EDX spectrum, it can be observed that the peaks of the Sb and Te elements have an approximate ratio of 2:3, indicating the stoichiometry of the Sb_2_Te_3_ film, as summarized in Table 1. As shown in Table 1, we found that the Te atomic composition decreased slightly to 57.4 (at %) as the annealing temperature increased to 350°C. This indicates that the post-annealing temperature did not have a significant effect on the atomic composition of the films, and these observations are in good agreement with previous works on Sb_2_Te_3_ thin films [16]. As a result, we believe that all the thin films are principally stoichiometric. In addition, we observed that the grain sizes of the films grew with increasing annealing temperature, as has been observed previously [31].

**Table 1:** **Calculated average grain sizes and atomic compositions of Sb2Te3 thin films at different annealing temperatures**

**Annealing temperature (°C)**	**FWHM B(°)**	**Bcos** ***θ***	**Average grain size D (nm)**	**EDX (atom %)**	
				**Sb**	**Te**
200	0.283	0.00150	88	40.2	59.8
250	0.254	0.00135	98	40.2	59.8
300	0.246	0.00131	101	41.8	58.2
320	0.190	0.00104	127	41.8	58.2
350	0.193	0.00100	129	42.6	57.4

**Figure 2:**
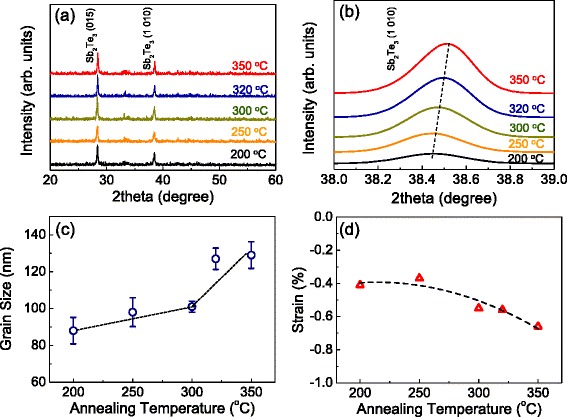
**XRD pattern, (1010) peak, and grain sizes and strains of Sb**_**2**_ **T**_**3**_ **thin films. (a)** XRD pattern and **(b)** (1010) peak of Sb2T3 thin films with increasing annealing temperatures of up to 350°C. **(c)** and **(d)** Grain sizes and strains of Sb_2_Te_3_ thin films as a function of post-annealing temperatures of up to 350°C, respectively.

The XRD patterns of the annealed Sb_2_Te_3_ films are shown in Figure 2a,b. From Figure 2a, it is apparent that two clear diffraction peaks are located at 28.24° and 38.29°, which are corresponding to the diffraction reflections of the (015) and (1010) planes of the Sb_2_Te_3_ films, respectively. A rhombohedral structure (JCPDS No. 71-393, $\mathrm{R}\overline{3}\mathrm{m}$) can be expected for the Sb_2_Te_3_ thin film, which is also consistent with the XRD patterns previously reported for Sb_2_Te_3_ films [16,26,32,33]. In addition, the XRD spectra show no further significant crystallinity changes when the annealing temperature is increased up to 350°C, as shown in Figure 2a, implying that the films obtained highly oriented crystalline structures under all annealing temperatures. The average grain size of the thin film was calculated using the Debye-Scherrer equation [34]

4 $$ D=k\lambda /\mathrm{Bcos}\theta, $$

where *k* is a constant (=0.89), *λ* is the wavelength of the radiation (=1.5401 Å), B is the full-width at half-maximum (FWHM), and *θ* is the diffraction angle from the XRD pattern. The detailed parameters of the Sb_2_Te_3_ thin films are summarized in Table 1. Using this method, we found the average grain sizes to be approximately 88 to approximately 129 nm for the films annealed at the temperatures of 200°C to 350°C, indicating that the grain sizes of the films increased as the annealing temperature increased, as shown in Figure 2c. This observation corresponds well with previous results for Sb_2_Te_3_ thin films [16]. To further investigate the effect of strain on the annealed Sb_2_Te_3_ thin films, we specially selected two main peaks, (015) and (1010) planes, and repeated XRD measurements in the vicinity of these peaks with a smaller 2*θ* interval (approximately 0.02), as illustrated in Figure 2b. We then found that the (1010) peak of the films shifted toward a much higher angle when the annealing temperatures were increased up to 350°C, as shown in Figure 2d, while the (015) peak did not reveal a shift for post-annealing temperatures of up to 350°C. This implies that the films experience a much larger compressive strain at this higher annealing temperature. The calculated compressive strain in the direction of (1010) was determined to be −0.39% to −0.62%. Further detailed effects of both the compressive strains and the grain sizes of the annealed thin films on the thermal transport will be discussed in the next section.

### Thermal properties of Sb_2_Te_3_ thin films

**Figure 3:**
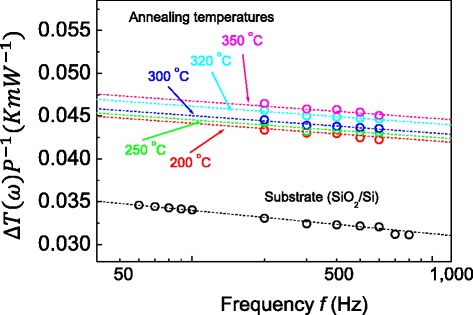
**Temperature oscillation of in-phase components for annealed Sb**_**2**_ **Te**_**3**_ **thin films.** Temperature oscillation of in-phase components for annealed Sb_2_Te_3_ thin films with annealing temperatures of 200°C, 250°C, 300°C, 320°C, and 350°C, as a function of applied frequency of up to 1,000 Hz. In addition, the temperature oscillation for the substrates is also included.

**Figure 4:**
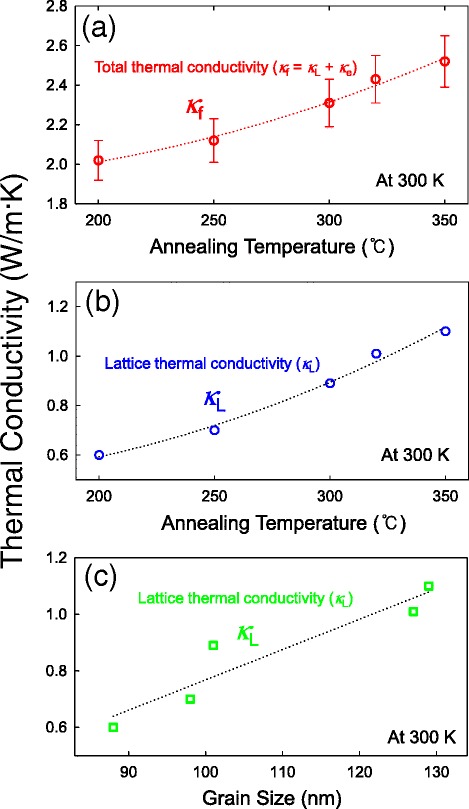
**Total and lattice thermal conductivities of the films at 300 K. (a)** Measured cross-plane (*κ*f) and **(b)** lattice thermal conductivity (*κ*L) of 400-nm-thick Sb2Te3 thin films at 300 K with different annealing temperatures of up to 350°C. **(c)** Measured lattice thermal conductivity of films as a function of grain size at 300 K. Dotted lines represent the best fitting.

Cross-plane thermal conductivity measurements were conducted using four-point-probe 3-*ω* measurements in the 20 to 300 K temperature range in a vacuum of 5 × 10^−5^ Torr, which has been proven to be a promising measurement technique for both 1D nanostructures [7,8,32] and 2D thin films [28]. Figure 3 shows the temperature oscillation of the in-phase component, Δ*T*_s+f_ (*ω*), for the 400-nm-thick Sb_2_Te_3_ thin films annealed at temperatures of 200°C to 350°C, with the Δ*T*_s_ (*ω*) value of the substrates (SiO_2_/Si) also added as a reference. As shown in Figure 3, we obtained a thermal conductivity for the SiO_2_ (300 nm thick)/Si substrate of approximately 1.25 W/m · K at 300 K, which is consistent with previous results [29]. Using Equation 3 and the slope of Figure 3, the cross-plane total thermal conductivity of the films at room temperature (as a function of annealing temperatures up to 350°C) were obtained from *κ*_*f*_ = *κ*_*e*_ + *κ*_*L*_, comprising both the electronic (*ĸ*_e_) and lattice (*ĸ*_*L*_) thermal conductivity components. Figure 4a,b shows the total and lattice thermal conductivities of the films at 300 K at annealing temperatures of up to 350°C. The average total thermal conductivity of the films was in the 2.02 to 2.52 W/m · K range at 300 K, which is approximately 1.6 to 2.0 times less than that of homogeneous Sb_2_Te_3_ single-crystal bulk materials at 300 K [19]. As shown in Figure 4a, the total thermal conductivity increased as the annealing temperature increased, indicating a similar trend to those of the grain size and compressive strain, as shown in Figure 2c,d. As a result, this increase in the total thermal conductivity of the films with increasing annealing temperature is clearly associated with the grain growth and strain of the films. This finding is very consistent with previous reports on the effects of nanostructure morphology and enhanced phonon scattering at the grain boundaries of thin films on the thermal conductivity [35-38]. Previous reports using numerical simulations demonstrated that the thermal conductivity generally increases with increasing compressive strain [25]. Note that Takashiri et al. reported that the dominant factors in the reduction of the *ĸ*_*L*_ of nanocrystalline bismuth antimony telluride thin films were the grain size and thin-film strain, here the grain size and strain were determined to be approximately 38 to approximately 93 nm and −0.8% to −1.4% while the *ĸ*_*L*_ were determined to be approximately 0.29 to approximately 0.39 W/m · K [35]. We found that these two components are determined to be approximately 88 to approximately 129 nm and −0.39% to −0.62%, while the *ĸ*_*L*_ of Sb_2_Te_3_ thin films was in the 0.61 to 1.08 W/m · K range at 300 K (Figure 4c). Hence, we expect that the reduction of the thermal conductivity of the Sb_2_Te_3_ thin films can be attributed to the combined grain-size and strain effects.

To estimate the electronic thermal conductivity using the Wiedemann-Franz law, *κ*_*e*_ = *LT*σ, where *L* is the Lorenz number (2.45 × 10^−8^ WΩ/K^2^) and *T* is the absolute temperature [19], it should be noted that our measurements for σ are for the in-plane electrical conductivity only, as mentioned previously in the experimental section. Therefore, to evaluate the out-of-plane electrical conductivity of the films, we estimated its value using anisotropic TE properties of layered Sb_2_Te_3_ bulk materials, where the relationship between the in-plane and out-of-plane electrical conductivities is *σ*_11_/*σ*_33_ = 1.8 [39]. Here, it was assumed that the electronic and thermal anisotropic properties of the single crystal bulk materials are the same as that of the highly crystalline thin film. Consequently, the estimated out-of-plane electrical conductivity of the films as a function of the annealing temperature was determined to be approximately 1,080 ± 55.1 to 2,833.9 ± 55.1 S/cm, as shown in Figure 4b. Figure 4c shows that the out-of-plane lattice thermal conductivity increases constantly with increasing grain size at 300 K and the thermal conductivity of the 400-nm-thick Sb_2_Te_3_ thin films as a function of the grain size indicates strong grain-size dependence of the thermal transport at 300 K.

### Theoretical modeling of temperature-dependent thermal conductivity of the films

**Figure 5:**
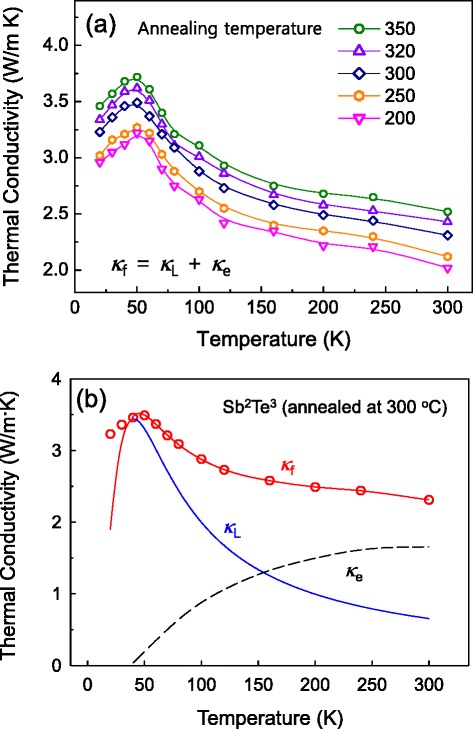
**Measured out-of-plane total thermal conductivity of the 400-nm-thick Sb**_**2**_ **Te**_**3**_ **thin films. (a)** Measured total thermal conductivity, *κ*f = *κ*L + *κ*e, of 400-nm-thick Sb2Te3 films annealed at temperatures of 200°C, 250°C, 300°C, 320°C, and 350°C as a function of temperature. **(b)** Measured total thermal conductivity of film annealed at 300°C as a function of temperature (red in scatter plot). For comparison, the theoretically calculated total thermal conductivity (*κ*_f_, solid line in red) with the two components of the thermal conductivity (the electronic (*κ*_e_, dotted line in black) and lattice thermal conductivities (*κ*_L_, solid line in blue)), are also included. *κ*_e_ was calculated from the out-of-plane electrical conductivity using the Wiedemann-Franz law.

Figure 5a shows the measured out-of-plane total thermal conductivity of the 400-nm-thick Sb_2_Te_3_ thin films as a function of temperature, from 20 to 300 K. As shown in Figure 5a, we found that the thermal conductivity of the films exhibits a strong dependence on the annealing temperature and grain-size in the 20 to 300 K temperature ranges. The observed reduction in the temperature-dependent total thermal conductivity was due to enhanced phonon boundary scattering following the decrease in grain size, as has been reported previously [16,35]. From Figure 5a, it can be seen that the total thermal conductivity decreases constantly after the approximately 50 K peak (the so-called ‘Umklapp peak’) has been reached, since the thin films at these temperatures are more significantly affected by phonon-phonon Umklapp scattering. For further understanding of lattice and electronic contribution in total thermal conductivity, we investigated a phonon transport model, which is based on the relaxation time and was previously predicted by Callaway in 1959 [40]. The details of this model are described elsewhere [40,41]. The expression for *κ*_L_ is given as follows [40]

5 $$ {\kappa}_L(T)=\frac{k_B}{2{\pi}^2c}{\left(\frac{k_BT}{\hslash}\right)}^3{\displaystyle {\int}_0^{\theta_D/T}{\tau}_c\frac{x^4{e}^x}{{\left({e}^x-1\right)}^2}\;dx,} $$

where *k*_B_ is the Boltzmann constant, *ℏ* is the reduced Planck constant, *x* is the dimensionless parameter with *x* = *ℏω*/*k*_*B*_*T*, *θ*_D_ is the Debye temperature, *T* is the absolute temperature, and *c* is the velocity of sound. Under the relaxation time approximation, we modify the scattering mechanism of the phonons, which were originally suggested by Callaway, and incorporate various scattering mechanisms including dislocation, point defects, phonon-phonon scattering, and boundary scattering from the grain and thickness effect. Thus, the total modified phonon scattering rate (relaxation time, *τ*_c_) is given by

6 $$ {\tau}_c^{-1}=\frac{c}{d_1}+\frac{c}{d_2}+A{\omega}^4+B{\omega}^2 Texp\left(-\frac{\theta_D}{3T}\right)+C\omega, $$

where *d*_1_ is the grain size of the thin films, as shown in Figure 2c and Table 1, *d*_2_ is the film thickness (400 nm), *c* (2,900 m/s) is average sound velocity from bulk Sb_2_Te_3_, and the coefficients *A*, *B*, and *C* are temperature-independent fitting parameters. In Equation 6, the first term, $\frac{c}{d}$, represents boundary scattering; the second term, *Aω*^4^, represents point-defect scattering; the third term, $B{\omega}^2 Texp\left(-\frac{\theta_D}{3T}\right)$, represents three-phonon Umklapp scattering, while the fourth term, *Cω*, represents carrier-phonon scattering. For the Sb_2_Te_3_ film, the relaxation-time fitting parameters *A*, *B*, and *C* are approximately 9.6 × 10^−43^ S^3^, approximately 2.7 × 10^−17^ S/K, and approximately 8.2 × 10^−5^, respectively, with the best fitting from the bulk Sb_2_Te_3_ [19]. Figure 5b shows the measured cross-thermal conductivity (*κ*_f_) of the Sb_2_Te_3_ thin film annealed at 300°C with the measured temperatures from 20 to 300 K, together with the theoretically calculated total thermal conductivity (solid-line) and calculated *κ*_e_ and *κ*_L_ values. As shown in Figure 5b, we found that the electronic contribution to the total thermal conductivity becomes more pronounced compared to that of the lattice component between temperatures of approximately 150 to 300 K, where the contribution of *κ*_e_ reaches approximately 72% at 300 K. Finally, Figure 5a,b shows that the temperature-dependent thermal conductivity of the Sb_2_Te_3_ thin films is strongly dependent on the grain size in the 20 to 300 K temperature ranges.

## Conclusions

In summary, we investigated the effect of strain and grain size on the thermal transport of Sb_2_Te_3_ thin films deposited on SiO_2_/Si substrates using the 3-*ω* technique. The measured total thermal conductivities of the Sb_2_Te_3_ thin films annealed at temperatures of 200°C, 250°C, 300°C, 320°C, and 350°C were determined to be 2.0 to 3.7 W/m · K in the 20 to 300 K temperature range. We then found that both grain size and strain have noticeable effects on the reduction of the total thermal conductivity of the Sb_2_Te_3_ thin films. The experimentally measured results for the thin films were also analyzed using a modified Callaway approach. Hence, we suggest that careful control of grain size or strain is the key to the development of high-performance TE devices.
